# Overcoming Obstacles During Caesarean Section with a Fibroid in the Uterus, from Diagnosis to Decision: A Case Series

**DOI:** 10.7759/cureus.39642

**Published:** 2023-05-29

**Authors:** Mukta Agarwal, Smita Singh, Shivangni Sinha, Upasna Sinha

**Affiliations:** 1 Obstetrics and Gynaecology, All India Institute of Medical Sciences Patna, Patna, IND; 2 Radiology, All India Institute of Medical Sciences Patna, Patna, IND

**Keywords:** caesarean section, myoma, myomectomy, pregnancy, fibroid

## Abstract

Background: We regularly face pregnancy with fibroid since pregnancy at advanced ages has been more prevalent and the prevalence of lower segment caesarean section (LSCS) has also increased over the previous three decades. Myomectomy with cesarean section has historically been avoided because of the danger of haemorrhage, but obstetricians now place more emphasis on it. Since fibroids can range widely in terms of location, size, and patient features, the intervention should be individualized. Under this article, we, therefore, provide a case series of seven pregnant women with uterine myomas who had delivery via LSCS.

Method: Seven pregnant patients who had uterine fibroid and undergone cesarean section were enrolled in this observational study done over the period of one year with consent and after taking ethical approval.

Results: The mean age was 27.7 years. Three of the cases were primigravida, while the remainder were multigravida. One patient had red degeneration and was hospitalized with abdominal discomfort at 29 weeks gestation. Four patients had a solitary fibroid, while the three had numerous. The biggest myoma size was 8×7 cm, while the smallest was 5×5 cm. Due to the presence of the fibroid in the lower segment of the uterus, three patients had a caesarean myomectomy, while in rest four cases it was not done. During cesarean myomectomy, two of them had uterine artery ligation to limit the moderate intraoperative haemorrhage.

Conclusion: If the patient is wisely chosen and the surgeon has the experience, a caesarean myomectomy can be performed safely and successfully during LSCS, especially if located in the lower uterine segment (LUS).

## Introduction

The most prevalent benign genital tumour in women is leiomyoma. During pregnancy, the frequency ranges between 1.6-10.7% [1]. However, due to the growing trend of delayed childbearing, the incidence of fibroids in older women undergoing treatment for infertility is reportedly 12-25% [2]. Myomectomy during the caesarean section has traditionally been avoided due to the danger of haemorrhage and postoperative morbidity, although numerous authors have demonstrated that myomectomy does not increase the risk of haemorrhage [3-7].

The bulk of fibroids (60-78%) do not increase in size during pregnancy, but one-third may do so in the first trimester, according to prospective research using ultrasound to monitor the size of the uterine fibroids [8]. Physical inspection can only detect big fibroids, and ultrasound's capacity to identify fibroids in pregnancy is even more limited due to the foetus [9]. Hence, the prevalence of uterine fibroids during pregnancy is underestimated [10].

Multiple (more than three) and large (greater than 5 cm) fibroids increase the risk of spontaneous miscarriage, preterm labour, placental abruption, premature rupture of membranes, foetal malpresentation, labour dystocia, caesarean delivery, postpartum haemorrhage (PPH), and hysterectomy [11]. They may induce foetal abnormalities (limb reduction, caudal dysplasia, head distortion, and congenital torticollis) if they are bigger than 10 cm [12]. Some studies also found a higher risk of intrauterine foetal death [13]. The pregnant lady may report excessive abdominal distension or intense pain brought on by myoma degeneration or torsion [14].

Because of the inherent hazards, fibroid in pregnancy necessitates risk stratification and case selection for myomectomy. In this case series, we provide the outcomes of seven patients who had fibroid tumours during pregnancy and had lower segment caesarean surgery.

## Materials and methods

In a tertiary care facility, this observational case series study was carried out with the participants' consent and with ethical committee approval over the duration of one year (AIIMS/Pat/IEC/2023/08). All who refused to participate were excluded. The study comprised seven pregnant women with uterine fibroid and, who underwent lower segment caesarean section (LSCS) delivery. The patients' medical background, including previous delivery methods, any history of abortion, history of any prior myomectomy, and the ultrasonographic location of myoma, were thoroughly recorded. The intraoperative location, number, and size of the fibroid were all documented. The use of blood transfusions and intraoperative bleeding, as well as its relation to myomectomy and foetal distress, were also studied.

## Results

This case series comprised seven pregnant women with fibroid who were delivered by caesarean section. The mean age was 27.7 years. Three of the cases were primigravida, while the remainder were multigravida. Two of them had a history of miscarriages. Three of the four multigravidas had a history of prior caesarean sections. Three of them were previously identified as having fibroid uteri before pregnancy, and four were diagnosed during pregnancy. One patient had undergone a myomectomy six years prior (Table 1).

**Table 1:** Patient Details G: Gravida; P: Parity; A: Abortion; L: Live issues; LSCS: Lower segment caesarean section

S. no.	Age	Obstetric score	Gestational age	History of abortion	Previous mode of delivery	Fibroid diagnosed before pregnancy	Fibroid diagnosed in pregnancy	History of myomectomy before pregnancy
1.	29	G1	39+3	-	-	Yes	-	-
2.	28	G5P1A3	37+3	3	LSCS	-	Yes	-
3.	33	G2P1L1	37+4	-	LSCS	Yes	-	Yes
4.	25	G1	38+1	-	-	-	Yes	-
5.	25	G2A1	38	1	-	-	Yes	-
6.	32	G1	37+4	-	-	-	Yes	-
7.	23	G2P1	36+5	-	LSCS	Yes	-	-

**Figure 1:**
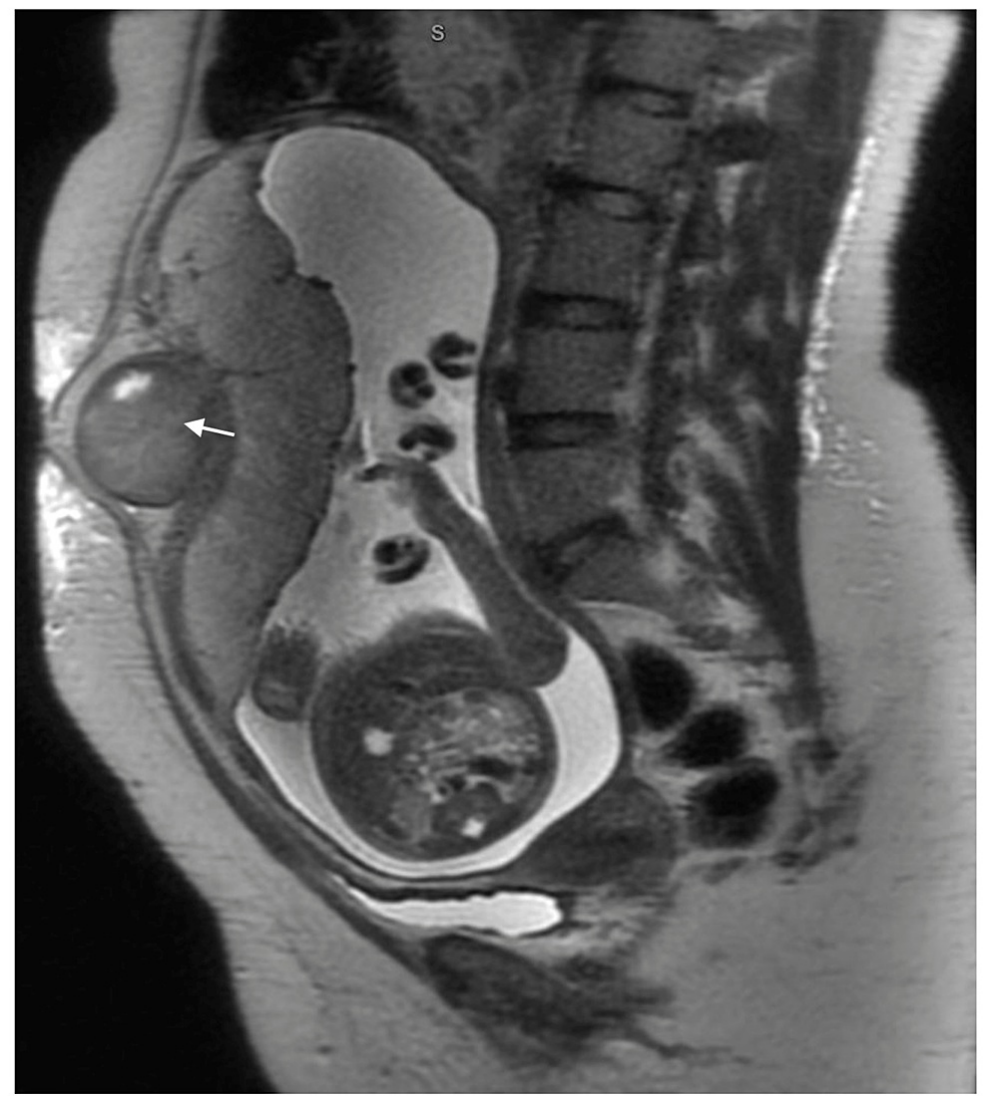
Sagittal T2 weighted MRI shows a sub-serosal fibroid with red degeneration along the anterior wall of the uterus in an upper uterine segment (white arrow)

One of them was hospitalized with abdominal discomfort at 29 weeks gestation. Red degeneration was identified on MRI and was treated cautiously (Figure 1).

 

**Figure 2:**
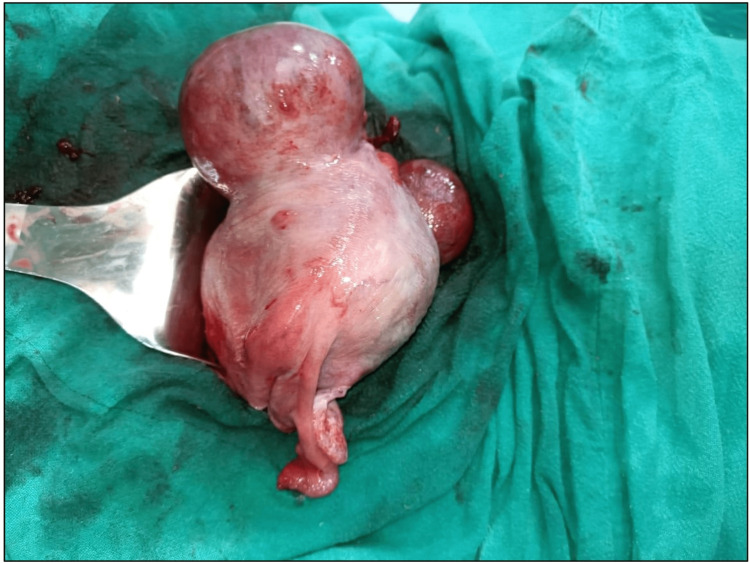
Multiple fibroids

**Figure 3:**
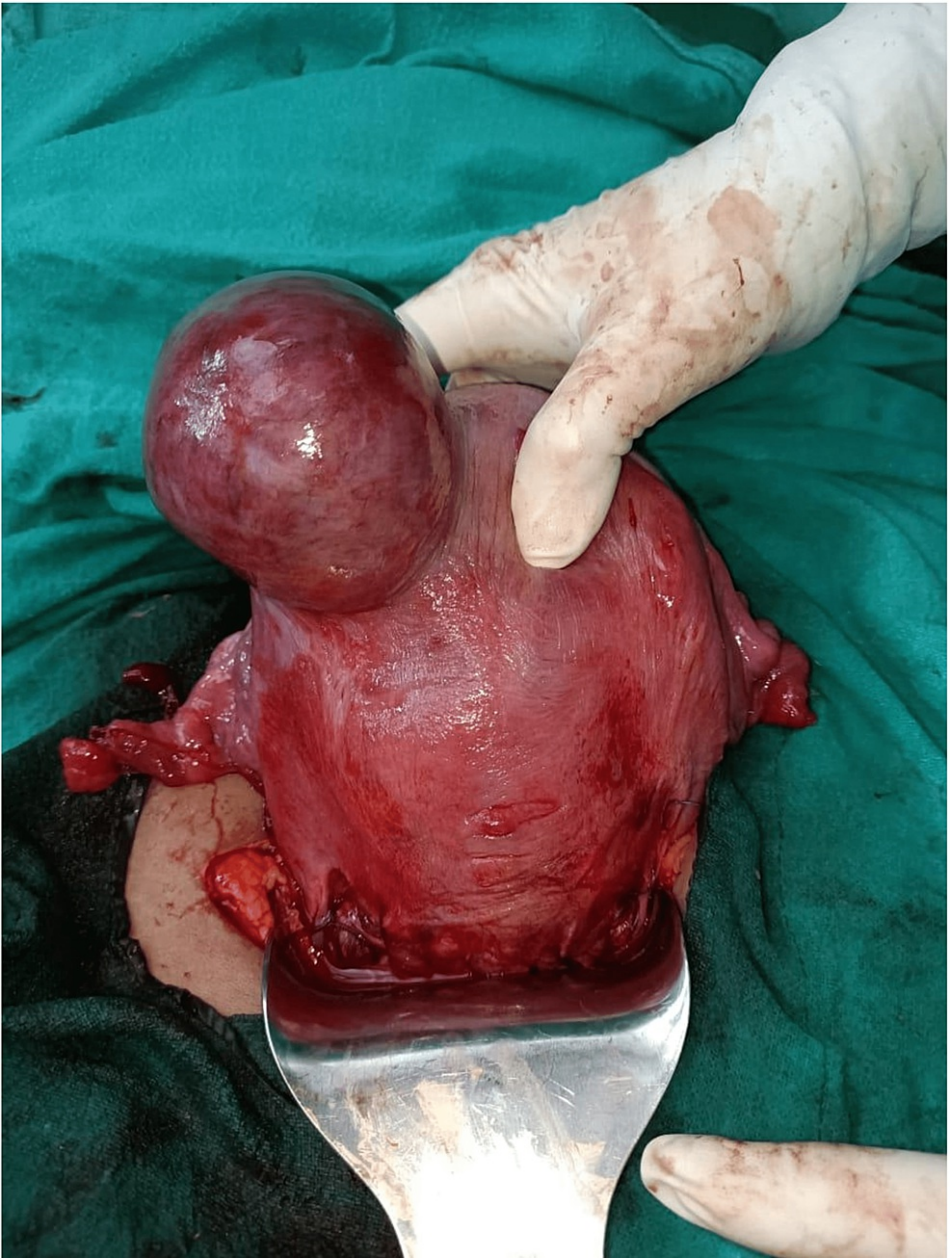
Big sub-serosal fibroid

Four patients had a solitary fibroid during surgery, while the others had numerous. The biggest myoma measured was 8×7 cm, while the tiniest measured 5×5 cm. In three of the instances, the tumour was in the lower uterine cavity. A caesarean myomectomy was performed in all three cases where the fibroid was in the lower uterine segment (LUS). In the remaining four cases, the myoma was in the upper segment, and none of them endured myomectomy (Figures 2,3).

 Myoma FIGO (International Federation of Obstetrics and Gynaecology) staging was FIGO-4, FIGO-6, FIGO-2, and FIGO-3 in three, two, one, and one patient, respectively (Table 2).

**Table 2:** Fibroid characteristics FIGO: International Federation of Obstetrics and Gynaecology; LUS: Lower uterine segment

No.	Number of fibroids	Size (cm)	Location	Degeneration	FIGO stage
1.	Single	5×5	Anterior wall	Red degeneration	FIGO 6
2.	Single	7×6	Anterior LUS	-	FIGO 3
3.	Multiple	8×7 (largest)	Anterior LUS	-	FIGO 4
4.	Single	7×6	Left LUS	-	FIGO 2
5.	Multiple	8×7 (largest)	Fundus	Fatty degeneration	FIGO 6
6.	Single	5×7	Posterolateral wall	-	FIGO 4
7.	Multiple	7×6	Right cornual	-	FIGO 4

There were four cephalic presentations, one breech presentation, and one compound presentation. Foetal distress was the most frequent (n = 3) indication for a caesarean section; in two cases, there was a myoma in the LUS with a history of previous LSCS; in one case, it was performed for malpresentation; and in the final case, there was a thin scar from a previous LSCS. Meconium-stained liquor was present intraoperatively in all cases of foetal distress. Intrapartum bleeding was mild in one and moderate in two of three caesarean myomectomy cases. In two instances, uterine artery ligation was performed in conjunction with uterotonics to limit haemorrhage. All four patients required blood transfusions. One patient experienced crepitations in the thorax on postoperative day one, and a chest X-ray revealed moderate pulmonary oedema. All other patient’s postoperative periods were uncomplicated, and they were discharged on Day 3 in stable condition (Table 3).

**Table 3:** Intraoperative Findings B/L: Bilateral; LUS: Lower uterine segment; PPH: Postpartum haemorrhage; MSL: Meconium-stained liquor; LSCS: Lower segment caesarean section

Sr. no.	Foetal presentation	Indication of LSCS	Intra-op	Caesarean myomectomy	Indication of caesarean myomectomy	PPH	Blood transfusion
1.	Cephalic	Foetal distress	MSL	No	-	No	No
2.	Cephalic	Previous LSCS with LUS fibroid	B/L uterine artery ligation	Yes	Fibroid over the incision site	Moderate	Yes
3.	Cephalic	Previous LSCS with LUS multiple fibroids	Uterotonics	Yes	Fibroid over the incision site	Mild	Yes
4.	Breech	Primi breech	Left uterine artery ligation	Yes	Fibroid over the incision site	Moderate	Yes
5.	Cephalic	Foetal distress	MSL	No	-	No	No
6.	Cephalic	Foetal distress	MSL	No	-	Mild	Yes
7.	Compound presentation	Previous LSCS with thin scar presentation	Adhesion	No	-	No	No

One baby was admitted to the neonatal intensive care unit (NICU) for meconium aspiration with an Appearance, Pulse, Grimace, Activity, and Respiration (APGAR) score of 7/10, kept on continuous positive airway pressure (CPAP) for 24 hours and later handed over to the mother. One baby had a low birth weight (Table 4).

**Table 4:** Baby Details APGAR: Appearance, pulse, grimace, activity, and respiration; NICU: Neonatal intensive care unit

	APGAR score	NICU admission	Reason for NICU admission	Low birth weight
1.	9/10	No	-	3.6 Kg
2.	9/10	No	-	2.5 kg
3.	9/10	No	-	3.1 kg
4.	7/10	Yes	Meconium aspiration	2.5 kg
5.	9/10	No	-	2.3 kg
6.	8/10	No	-	2.8 kg
7.	8/10	No	-	2.8 kg

## Discussion

Several studies have produced contradictory results about the impact of fibroid on pregnancy, its difficulties, feto-maternal morbidity, and removal through caesarean section. According to studies, 10-30% of pregnant women with myoma have complications because of the presence of these tumours [1].

In the present case series, the average age was 27.7 years: The minimum age was 23 and the maximum was 33. Fibroids were discovered before pregnancy in three out of seven instances, and two patients had a history of abortions. Radhika et al. conducted a prospective study of the maternal and foetal outcomes in 15 women who were pregnant with uterine fibroids. Most patients with fibroids were in the 25-30 age group, as opposed to the 31-35 age group (66% vs 33%) [16]. Comparing multi-gravidae to primigravidae, fibroids were more common in the latter group. Fibroids were discovered before pregnancy in nearly half of the individuals (53.3%). Three (20%) of the 15 women had abortions. Eleven out of 12 patients achieved term pregnancy between 37 and 40 weeks. 75% of women who had a full-term pregnancy underwent a caesarean section. Five out of 15 (33.3%) individuals experienced PPH. Five of 12 kids were born with low birth weights. Four newborns were admitted to the NICU. Their investigation discovered a more vital link between fibroid and multigravida patients than we did in our study. Our findings are likewise congruent with the findings of this investigation [15,16].

Three of the seven patients had fibroids before conception, two of them with multiple fibroids and one with a single fibroid. There was no growth or decrease in myoma size in any of them. In contrast with Benaglia et al., who, in their prospective cohort research on 25 women with fibroids, reported that the sizes of the fibroids dramatically increased to more than double their original sizes during the first seven weeks of pregnancy, Tîrnovanu et al. observed change in the maximal rise occurred in the second trimester [10,17]. Nevertheless, Rosati et al. discovered that up to 78% of uterine fibroids do not demonstrate significant growth during pregnancy [18].

In our study, foetal distress was the most prevalent reason for a caesarean section. This is consistent with the findings of Noor et al., who found that 38% of caesarean sections were performed due to foetal distress [19]. We discovered a solitary fibroid in four out of seven individuals, with the majority of them localized in the anterior LUS. Two patients had no complications, but in four cases, PPH occurred, malpresentation in two cases, premature rupture of membrane in one case, and myomectomy was done in three cases due to its location over LUS, which is consistent with several studies [20-22]. Posh et al.'s research showed two NICU admissions, consistent with this study in which one infant was admitted to the NICU [1].

## Conclusions

Fibroid pregnancy is not rare these days, since the average age of marriage and pregnancy has climbed in the last two decades. Although research has shown that fibroid in pregnancy can cause a variety of antepartum and intrapartum difficulties, myomectomy during caesarean delivery has long been debated. Our case series demonstrates that a selective case decision with a team effort is essential to address the fibroids complicating pregnancy, whether it be PPH, premature labour, or myomectomy. However, the study is limited to a small sample size, so there is a need for further research involving larger cohorts to enhance the generalizability of the findings.
